# Rapid evolution of the PB1-F2 virulence protein expressed by human seasonal H3N2 influenza viruses reduces inflammatory responses to infection

**DOI:** 10.1186/s12985-017-0827-0

**Published:** 2017-08-22

**Authors:** Julie McAuley, Yi-Mo Deng, Brad Gilbertson, Charley Mackenzie-Kludas, Ian Barr, Lorena Brown

**Affiliations:** 1 Department of Microbiology and Immunology, University of Melbourne at the Peter Doherty Institute for Infection and Immunity, 792 Elizabeth St, Melbourne, VIC 3000 Australia; 2 WHO Collaborating Centre for Reference and Research on Influenza (WHO-CCRRI) at the Peter Doherty Institute for Infection and Immunity, Victoria, Australia

**Keywords:** PB1-F2, Influenza A virus, Pandemic, Seasonal, Inflammation, Virulence, Pathogenicity, Respiratory disease

## Abstract

Influenza A virus (IAV) PB1-F2 protein has been linked to viral virulence. Strains of the H3N2 subtype historically express full-length PB1-F2 proteins but during the 2010–2011 influenza seasons, nearly half of the circulating H3N2 IAVs encoded truncated PB1-F2 protein. Using a panel of reverse engineered H3N2 IAVs differing only in the origin of the PB1 gene segment, we found that only the virus encoding the avian-derived 1968 PB1 gene matching the human pandemic strain enhanced cellular infiltrate into the alveolar spaces of infected mice. We linked this phenomenon to expression of full-length PB1-F2 protein encompassing critical “inflammatory” residues.

**Electronic supplementary material:**

The online version of this article (doi:10.1186/s12985-017-0827-0) contains supplementary material, which is available to authorized users.

## Background

The 1968 influenza pandemic, caused by the newly emerged H3N2 virus, was associated with 3 million deaths. Since then the H3N2 IAV has become endemic in the human population but remains a major cause of seasonal influenza epidemics. In contrast, illness caused by the recent 2009 H1N1 pandemic was, for most infected individuals, no worse than a seasonal influenza infection and the resulting death toll was estimated at <500,000 [1]. When the 1968 H3N2 virus first infected humans, the RNA gene segment encoding the polymerase subunit PB1 was derived from an avian virus precursor [2], while the PB1 gene segment from the 2009 H1N1 pandemic IAV was derived from a circulating human H3N2 strain [3]. Thus, the avian origin of PB1 and the accessory proteins it encodes may have contributed to the severity of disease during the 1968 pandemic.

The PB1-F2 protein is encoded by a + 1 alternate reading frame of the PB1 gene [4]. PB1-F2 is encoded by nearly all IAV isolates, however, since 1947 nearly all circulating human isolates of the H1N1 subtype have evolved to express a truncated PB1-F2 of 57 amino acids (AA) [5]. The 2009 H1N1 pandemic virus coded for an 11 AA truncated form of PB1-F2 protein, which is assumed to be non-functional [3]. Truncated PB1-F2 of 34 amino acids in circulating human H3N2 IAVs have recently been detected in the USA, Europe [6, 7] and Asia [8]. In contrast to other avian IAVs, a high proportion of recent isolates of highly pathogenic H5N1 influenza viruses have also been found to have mutations in PB1-F2 leading to truncation of the protein [9].

Full genome studies on H3N2 IAVs isolated between 1968 and 2011 have revealed that rates of amino acid substitution are substantially higher for PB1-F2 than for PB1 [6, 7]. Taking this into consideration, we hypothesized that the evolution of PB1-F2 might confer some fitness advantage for the H3N2 virus during the course of mammalian infection and/or aid evasion of host-immunological responses. In this study we used reverse genetics to create a panel of H3N2 viruses that differed only in the origin of their PB1 gene, as well as the corresponding viruses that expressed truncation mutants of PB1-F2, to investigate the specific functional role of PB1-F2 in H3N2 virus evolution.

## Methods

### Sequence analysis of PB1-F2 protein of H3N2 IAV

The gene sequences encoding PB1 derived from H3N2 IAV isolates were downloaded from the Global Initiative on Sharing All Influenza Data platform (www.gisaid.org). These data were supplemented with an additional 303 PB1 gene sequences from Australian virus isolates submitted to the WHO-CCRRI, Victoria, Australia. These viruses were grown in Madin Darby canine kidney (MDCK) cells, then sequenced and analyzed as described previously [10]. The predicted AA sequence of the PB1-F2 proteins from the different isolates were generated by analysis of the +1 alternative open reading frame (ORF) of the PB1 gene, which begins 92 nucleotides downstream of the first ATG start codon of the PB1 ORF.

### Generation of viruses

A set of plasmids were generated on the pHW2000 backbone which encoded the HA, NA, PB2, NP, PA, M and NS gene segments of the H3N2 virus A/Udorn/307/1972. To create a panel of viruses differing only in the (H3N2) PB1 gene segment, additional plasmids were generated to express the PB1 segment of A/Hong Kong/1/1968 (genbank ID: AF348172), A/Udorn/307/1972 (genbank ID: CY009642), A/Panama/2007/1999 (genbank ID: DQ487333), and A/Perth/10/2010 (genbank ID: CY121502). To create truncated PB1-F2 viruses, we utilized the Quikchange II Mutagenesis kit (Agilent Technologies) to alter the PB1-F2 ORF to encode a stop codon after 34 AA residues (T222A by Udorn PB1 nucleotide numbering) and mutated downstream start codons (T234C, T255C and T270C), so that translation did not re-initiate. Conversion of the truncated 2010 PB1-F2 ORF to encode a full-length 90AA protein involved a single point mutation (A198C). In no case did the mutations in the PB1-F2 ORF cause non-synonymous mutations in the PB1 ORF. The start codon of N40, another product of the PB1 gene, was intact in all PB1 plasmids and it is expected that N40 expression levels during virus infection remain unchanged. H3N2 viruses differing only in the origin of their PB1 gene, with and without the corresponding truncation mutation in PB1-F2, were then created by reverse genetics and contained all other gene segments from A/Udorn/307/1972. Viruses were rescued by one passage in MDCK cells, then propagated in eggs. All viruses were sequenced to ensure no inadvertent mutations occurred, then characterized as described [11]. Herein viruses containing genetic material from PB1 of a particular isolate are referred to by the isolate year.

### Animal model

Eight to ten week old female BALB/c mice were maintained in the Specific Pathogen Free Bioresources Facility at the Department of Microbiology and Immunology (University of Melbourne). Infectious agents were diluted in sterile PBS and 50 μL administered intranasally to anaesthetized mice (inhaled 2.5% isofluorane; Baxter Healthcare). Mice were monitored daily for weight loss and illness. The infectious dose for all experiments was 100 plaque-forming units (PFU). At 72 h post-infection (hpi), bronchioalveolar lavage (BAL) fluid and lung samples were extracted for analysis of cellular content and viral load. Viral RNA from these samples was sequenced to confirm no further mutations had occurred in vivo. Viral load was assessed via homogenization of whole lung tissue and determined by the quantitation of plaques on confluent MDCK cell monolayers as previously described [12].

### Assessment of the cellular and cytokine composition of bronchioalveolar lavage (BAL)

BAL was processed as previously described [11]. Briefly, leukocytes (CD45^+^), neutrophils (CD45^+^Ly6G^+^F480^−^) and alveolar macrophages (CD45^+^Ly6G^−^F480^+^MHCII^int^) were enumerated by flow cytometry and expressed as a proportion of cellular events analyzed. Total numbers of each cell population were calculated in relation to the number of white blood cells per mL (WBC/mL) in the original sample, determined by counting cells using the Trypan Blue exclusion method.

### Statistical analysis

Statistical analyses were performed using GraphPad Prism v6. All graphs show mean ± SE and are representative of at least two independent experiments.

## Results

The prototype H3N2 pandemic isolate A/Hong Kong/1/68 encoded a PB1-F2 protein of 90 AA in length. Our analysis of H3N2 PB1 sequences deposited in the GISAID database between 1968 and 2013 revealed that the 90 AA PB1-F2 is the dominant form of this predicted protein in circulation (80%) (Table 1). However, a significant number of H3N2 viruses (16.7%) were found to carry a truncated PB1-F2 of 34 AA. Prior to 1999, less than 2% of isolates had this truncated form of PB1-F2 but this percentage increased to 13% during 2000–2009, peaked at 55% in 2011, then decreased to 5% in 2013. The same pattern was observed in the subset of H3N2 viruses isolated in Australia (data included within the table). A small number of H3N2 virus isolates encoded PB1-F2 of other lengths, e.g. 87 and 79 AA, but disappeared quickly, suggesting they did not offer any advantage for the virus.

**Table 1:** Presence of full-length and various truncated forms of PB1-F2 in H3N2 viruses isolated in different years

Year of isolation	Total no. isolates	No. isolates with indicated PB1-F2 amino acid sequence length							
		10–27	34	57–76	79–81	87	90	95	101
Pre-1999	683	3	12 (1.8%)^a^	19	12	10	601 (88.0%)	0	26
2000–2009	2291	4	302 (13.2%)	7	20	40	1916 (83.6%)	1	1
2010	408	1	191 (46.8%)	1	0	0	215 (52.7%)	0	0
2011	330	1	182 (55.2%)	0	3	0	144 (43.6%)	0	0
2012	501	1	63 (12.6%)	1	2	0	434 (86.6%)	0	0
2013	396	0	20 (5%)	0	0	2	374 (94.4%)	0	0
All samples	4609	10	770 (16.7%)	28	37	52	3684 (80%)	1	27

^a^percentage of total isolates in that time span is shown for the dominant length PB1-F2 proteins

Using reverse engineering, we created a panel of viruses that were based upon seven gene-segments of H3N2 A/Udorn/307/1972 influenza virus, and contained the PB1 gene segment obtained from one of the following H3N2 influenza isolates: the pandemic virus A/Hong Kong/1/1968, A/Udorn/307/1972 and A/Panama/2007/1999, all of which encode a full-length PB1-F2 protein of 90 AA. We also included a virus expressing the PB1 of A/Perth/10/2010, which naturally codes for a 34 AA truncated PB1-F2 protein (Additional file 1: Figure S1). To investigate the effects of truncation of the PB1-F2 protein, the 1968, 1972, and 1999 PB1-encoding plasmids were genetically altered to insert a truncation mutation to match the 2010 PB1-F2 isolate length of 34 AA. In addition, the 2010 PB1 plasmid was mutated to encode a 90 AA PB1-F2 protein (Additional file 1: Figure S1). Upon characterizing each virus, all were found to grow to similar titers and analysis of viral replication kinetics after infecting human alveolar type II epithelial cells revealed similar rates of progeny production, indicating no loss to viral fitness (Additional file 2: Figure S2, *p* > 0.05, repeated measures ANOVA; Additional file 3: Methods).

**Fig. 1:**
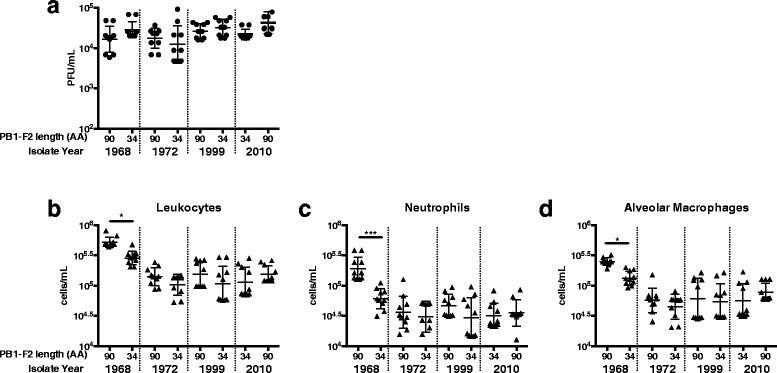
Full length PB1-F2 protein matching the 1968 pandemic isolate contributes to enhancement of inflammatory response to infection. BALB/cJ mice (*n* = 10, female, 8-10wk) were infected with 100 PFU reverse engineered A/Udorn/307/1972 virus containing the PB1 gene of the 1968, 1972, 1999 and 2010 isolates, or matched viruses genetically altered to produce 34 AA, or in the case of the 2010 strain a 90 AA, PB1-F2 protein. **a**) Viral load (PFU/mL) of homogenised lung tissue taken at 72 hpi was not significantly different between groups (*p* > 0.05, one-way ANOVA). BAL samples were taken at 72 hpi and examined for **b** leukocyte, **c** alveolar macrophage and **d** neutrophil content. * *p* < 0.05, *** *p* < 0.001 One-way ANOVA, Sidak’s multiple comparison test

To examine the contribution of PB1-F2 to enhancement of immunopathology in the context of a mild infection, we infected mice with our panel of H3N2 IAV differing only in their expression of PB1-F2/PB1 protein(s). At 72 hpi no significant differences were observed in viral lung titres (Fig. 1a) or weight loss (data not shown). Despite this, the numbers of leukocytes, alveolar macrophages and neutrophils in the BAL of mice infected with virus expressing wild-type PB1 from the 1968 isolate was significantly greater than those infected with virus expressing PB1 from the 1972 to 2010 isolates (Fig. 1). Importantly, infection of mice with virus producing the 1968 PB1 protein, but a truncated 34 AA PB1-F2 protein, showed significantly reduced cellular infiltrate compared to infection with virus expressing the unaltered 1968 PB1/PB1-F2 proteins, implicating the PB1-F2 protein in mediating the heightened innate cellular response. The levels of cellular infiltrates in mice infected with virus expressing the truncated 1968 PB1-F2 were equivalent to mice infected with virus expressing PB1 from the later virus isolates, regardles of PB1-F2 length (Fig. 1b-d).

## Discussion

Viruses causing the influenza H3N2 pandemic were first isolated in Hong Kong in July 1968. These viruses had a novel HA and PB1 derived directly from the wild aquatic bird virus reservoir, but shared the remaining gene segments with previously circulating H2N2 viruses [2]. Murine studies implicating PB1-F2 as a viral virulence factor have utilized either highly virulent viruses [9], or mouse-adapted virulent viruses (such as A/Puerto Rico/8/34 and the reassortant X31) [11, 13]. Here, we utilized a mouse-adapted H3N2 virus that induces mild symptoms that can be compared to low levels of disease of seasonal infection in humans. We provide evidence that the PB1 gene and the encoded avian PB1-F2 protein constitute virulence factors that enhance the mammalian cellular responses to infection in what would be an otherwise mildly-inflammatory virus.

As all the engineered viruses replicated to a similar extent irrespective of the PB1 they expressed, the differences in the levels of cellular infiltrate into the alveolar spaces could not be attributed to alterations in viral replication. Recently a study utilizing two variants of the mouse-adapted H1N1 PR8 virus revealed specific PB1 amino acid substitutions (E398G, S524G and I563R) that led to increased virulence of the virus and modulation of the host-antiviral IFN response [14]. However, none of the four different PB1 proteins from the IAV isolates examined here contain these AA substitutions and so these changes cannot account for the differences in inflammation observed in our infection model. Furthermore, while viruses expressing the PB1 matching the 1968 pandemic isolate revealed an increase in the cellular recruitment to the lungs, truncation of PB1-F2 significantly reduced the influx of leukocytes into the alveolar spaces, confirming that the enhanced inflammatory response was due to PB1-F2 and not additional sequences within PB1.

Full genome studies on H3N2 IAVs isolated between 1968 and 2011 have revealed that rates of amino acid substitution are substantially higher for PB1-F2 than for PB1 [6, 7] and some of these changes may have been responsible for the evolution away from an inflammatory phenotype prior to truncation. To date, 5 specific AA markers within the C-terminal region of the PB1-F2 protein have been linked to inducing inflammation [15, 16]. None of the H3N2 PB1 gene segments used here code for the S66 virulence marker [16]. The other markers, L62, R75, R79 and L82 [15], were defined by comparing inflammatory responses of mice to peptides matching the C-terminal third (AA 61–87) of the 1968 PB1-F2 with those to the closely related but non-inflammatory peptide from a 1995 H3N2 strain. Our experiments described here with virally-expressed PB1-F2 showed that loss of just one of these residues, L82, in the 1972 PB1-F2 was sufficient to reduce the leukocyte infiltration into the lungs to background levels seen with a virus expressing a truncated PB1-F2. The previous study investigating AA changes at each of the 4 inflammatory markers concluded that all 4 amino acids contributed to the proinflammatory phenotype in an H3N2 background [15]. This may explain the lack of enhanced cellular recruitment to the lungs during infection with H3N2 virus that encoded a PB1-F2 expressing only 3 of the 4 AA inflammatory markers. Alternatively or additionally, these data may highlight the critical importance of L82 over the other markers. In the previous peptide studies [15] an L82S substitution in the 1968 peptide or an S82 L substitution in the 1995 peptide showed the greatest effect on altering weight loss in mice to that of the opposing wildtype peptide.

Additional evidence of a selective pressure for circulating human H3N2 viruses to evolve away from producing a highly inflammatory PB1-F2 is that none of the inflammatory markers are present in the 1999 PB1-F2 ORF or when the ORF of the 2010 PB1-F2 is altered to code for a full-length protein. This again supports the importance of sequence rather than length as being critical to the inflammatory phenotype. We have previously shown that PB1-F2 peptides derived from the C-terminus of X-31 PB1-F2 potently induce cellular inflammatory responses through activation of the NLRP3 inflammasome and it is the aggregated fraction that is responsible [13]. While X-31 is an H3N2 virus, it expresses the internal genes, including the PB1-F2 gene, of the H1N1 PR8 strain. As the 1968 PB1-F2 C-terminal domain contains all 4 inflammatory residues and has been shown to stimulate inflammation, we propose that virus encoding the full-length 1968 H3N2 PB1-F2 protein also has the correct sequence to form aggregates when produced inside an infected cell, driving pattern recognition responses that lead to increased inflammation. The lack of enhanced cellular responses to H3N2 virus expressing the 1972 PB1-F2, may indicate that loss of the inflammatory marker L82 through drift results in an inability of the protein to form aggregates when produced inside the cell and is a current focus of our continued investigations.

While the majority of the current seasonal H3N2 viruses do not express truncated PB1-F2 proteins, the PB1 gene segment from the 2009 H1N1 pandemic IAV was derived from a circulating human H3N2 strain that produces a truncated PB1-F2 of 11AA and is a feature that continues to dominate currently circulating H1N1 IAVs [3]. As both virus subtypes induce minimal inflammatory disease during infection of otherwise healthy individuals, our data suggests both mutation of inflammatory residues and elimination of the C-terminal domain of the PB1-F2 protein, may be an important mechanism by which IAV escapes immunological detection, although we and others have not yet provided direct evidence of a selective advantage on the basis of a non-inflammatory PB1-F2.

Taken together, these results indicate direct derivation of the PB1 gene segment from the avian reservoir into novel IAV may encode a PB1-F2 protein that augments cellular inflammatory responses to infection in mammals. Subsequent evolution in humans within the first four years after emergence of the H3N2 viruses appeared to firstly encode a less inflammatory PB1-F2 protein by mutation of a major inflammatory marker, prior to loss of additional markers and the appearance of truncation mutations. We believe that the truncation offers no additional selective advantage over mutation of inflammatory markers for the virus to escape overwhelming innate defenses, and as such the current seasonal H3N2 IAVs circulating in humans are not dominated by strains encoding truncated forms of the PB1-F2 protein.

## Additional files

Additional file 1: **Figure S1.** Comparison of the predicted amino acid sequence of PB1-F2 proteins expressed by H3N2 viruses used in this study. After translation of the +1 ORF of the respective PB1 gene segments, the predicted amino acid sequence of the PB1-F2 of each isolate was aligned using Vector NTI. 2010. ∆ shows predicted amino acid sequence after the stop codon was mutated to encode serine at that position and a full-length 90 amino acid PB1-F2 protein was predicted to be produced. Shading highlights the differences in the amino acid sequences at that site (Black: 100% identical, Grey: Majority of sites identical, White: Minority of sites identical). Grey text indicates a unique amino acid. *L62, R75, R79 and L82 are predictive markers for enhancement of inflammation [15]. #S66 is a linked virulence determinant (none of the selected viruses carry this mutation) [16]. (DOC 30 kb)

Additional file 2: **Figure S2.** Viral replication kinetics of H3N2 viruses containing different PB1-F2/PB1 s in A549 cells. Confluent monolayers of A549 cells were infected with 0.03 MOI H3N2 A/Udorn/307/1972 virus containing wild-type PB1 or PB1 with genetically modified PB1-F2 of the i) 1968, ii) 1972, iii) 1999 or iv) 2010 isolates. At the time-points indicated, the supernatant and cells were harvested and evaluated for A) percentage of cells infected (NP-FITC^+^), B) amount of viral NP produced within an infected cell (MFI) and C) viral content (PFU/mL) (PDF 42 kb)

Additional file 3: Methods. Cell lines and viral infections [12, 17]. (DOC 26 kb)

## Abbreviations

AA: Amino acids
BAL: Bronchoalveolar lavage
hpi: Hours post infection
MDCK: Madin Darby canine kidney
ORF: Open reading frame
PFU: Plaque-forming units
WHO-CCRRI: WHO Collaborating Centre for Reference and Research on Influenza
