# Supplementary material for: Rapid evolution of the PB1-F2 virulence protein expressed by human seasonal H3N2 influenza viruses reduces inflammatory responses to infection
Source: Virol J. 2017 Aug 22;14:162. doi: 10.1186/s12985-017-0827-0 (PMC5568198; doi:10.1186/s12985-017-0827-0)
Supplement: Supplementary file 1 — Figure S1. Comparison of the predicted amino acid sequence of PB1-F2 proteins expressed by H3N2 viruses used in this study. After translation of the +1 ORF of the respective PB1 gene segments, the predicted amino acid sequence of the PB1-F2 of each isolate was aligned using Vector NTI. 2010. ∆ shows predicted amino acid sequence after the stop codon was mutated to encode serine at that position and a full-length 90 amino acid PB1-F2 protein was predicted to be produced. Shading highlights the differences in the amino acid sequences at that site (Black: 100% identical, Grey: Majority of sites identical, White: Minority of sites identical). Grey text indicates a unique amino acid. *L62, R75, R79 and L82 are predictive markers for enhancement of inflammation [15]. #S66 is a linked virulence determinant (none of the selected viruses carry this mutation) [16]. (DOC 30 kb) [file 12985_2017_827_MOESM1_ESM.doc]

**Additional file 1: Figure S1**

**1 34 * # * * * 91**

**1968 MEQEQDTPWTQSTEHINIQKKGSGQQTRKLERPNLTQLMDHYLRIMSQVDMHKQTVSWKQWLSLKNPTQGSLKTRVLKRWKLFNKQGWTD-**

**1972 MEQEQDTPWTQSTEHINIQKKGSGQQTQKLGRPNLTQLMDHYLRIMSQVDMHKQTVSWKQWLSLKNPTQGSLKTRALKRWKSFNKQGWTD-**

**1999 MEQEQGTPWTQSTEHTNIQKRGSGRQIQKLGHPNSTQLMDHYLRIMSQVDMHKQTVSWRLWPSLKNPTQGSLRAHALKQWKSFNKQGWTN-**

**2010 MEQGQGTPWTQSTEHTNIQRGGSGRQIQKLGHPN---------------------------------------------------------**

**2010∆ MEQGQGTPWTQSTEHTNIQRGGSGRQIQKLGHPNSTQLMDHYLRIMNQVDMHKQTVSWRLWPSLKNPTQVSLRTHALKQWKPFNRQGWTN-**
