# Supplementary figures and images for: Rapid evolution of the PB1-F2 virulence protein expressed by human seasonal H3N2 influenza viruses reduces inflammatory responses to infection
Source: Virol J. 2017 Aug 22;14:162. doi: 10.1186/s12985-017-0827-0 (PMC5568198; doi:10.1186/s12985-017-0827-0)

**A**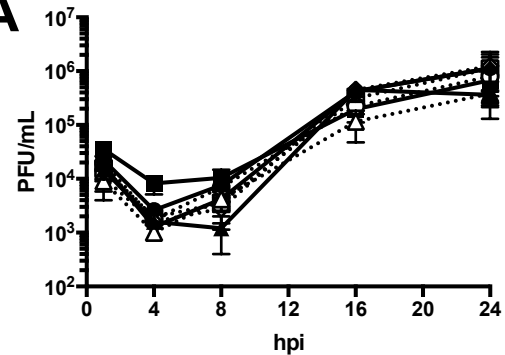

| Virus | Length of PB1-F2 (AA) |    |
|-------|-----------------------|----|
|       | 90                    | 34 |
| 1968  | ▲                     | △  |
| 1972  | ◆                     | ◇  |
| 1999  | ●                     | ○  |
| 2010  | ■                     | □  |

**B**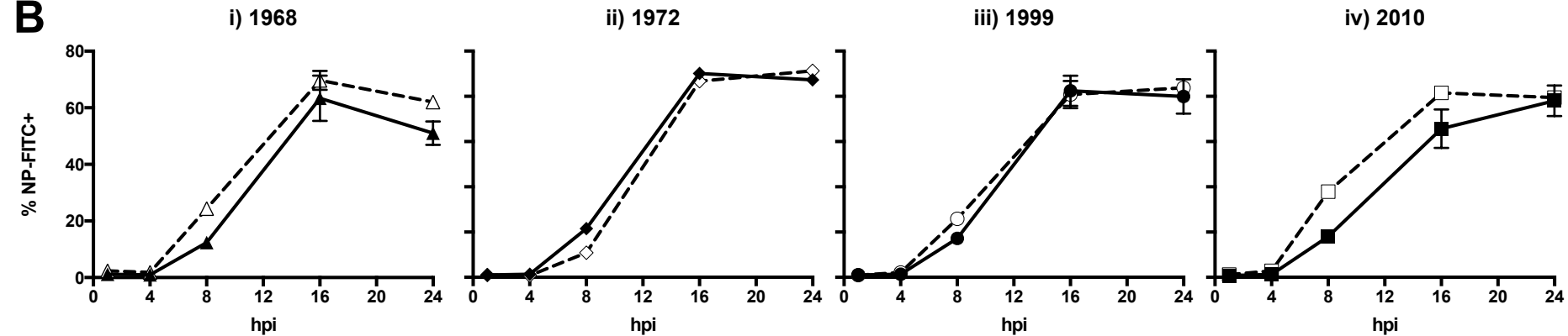

Supplement: Supplementary file 2 — Figure S2. Viral replication kinetics of H3N2 viruses containing different PB1-F2/PB1 s in A549 cells. Confluent monolayers of A549 cells were infected with 0.03 MOI H3N2 A/Udorn/307/1972 virus containing wild-type PB1 or PB1 with genetically modified PB1-F2 of the i) 1968, ii) 1972, iii) 1999 or iv) 2010 isolates. At the time-points indicated, the supernatant and cells were harvested and evaluated for A) percentage of cells infected (NP-FITC+), B) amount of viral NP produced within an infected cell (MFI) and C) viral content (PFU/mL) (PDF 42 kb) [file 12985_2017_827_MOESM2_ESM.pdf]
