# Supplementary material for: Rapid evolution of the PB1-F2 virulence protein expressed by human seasonal H3N2 influenza viruses reduces inflammatory responses to infection
Source: Virol J. 2017 Aug 22;14:162. doi: 10.1186/s12985-017-0827-0 (PMC5568198; doi:10.1186/s12985-017-0827-0)
Supplement: Supplementary file 3 — Methods. Cell lines and viral infections [12, 17]. (DOC 26 kb) [file 12985_2017_827_MOESM3_ESM.doc]

Additional file 3

MDCK and A549 cells were grown in RPMI-1640 medium supplemented with 10% heat inactivated fetal bovine serum (FBS), 2mM glutamine and antibiotics. Cell cultures were maintained at 37°C in a 5% CO_2_ incubator. Cells were washed once with phosphate buffered saline (PBS), infected with virus at the indicated multiplicity of infection (MOI) and further incubated as described previously [17]. Cell culture supernatants were harvested at various times post-infection as specified. The infectious viral titer was determined by the quantitation of plaques on confluent MDCK cell monolayers as previously described [12].
